# Molecular epidemiology and emergence of sequence type 25 hypervirulent Klebsiella pneumoniae in pigs in the Netherlands (2013–2020): a global comparative analysis with human and pig isolates

**DOI:** 10.1099/mgen.0.001388

**Published:** 2025-04-23

**Authors:** Soe Yu Naing, Aldert Zomer, Linda van der Graaf-van Bloois, Manon Houben, Karin Junker, Otto Schreurs, Annet Heuvelink, Jaap A. Wagenaar, Jobke van Hout

**Affiliations:** 1 Department of Biomolecular Health Sciences, Faculty of Veterinary Medicine, Utrecht University, Utrecht, Netherlands; 2 Royal GD, Deventer, Netherlands; 3 Advee Dierenartsen, Ysselsteyn, Netherlands; 4 Wageningen Bioveterinary Research, Wageningen University & Research, Lelystad, Netherlands

**Keywords:** aerobactin plasmid, antimicrobial resistance, emerging infectious diseases, hypervirulent *Klebsiella pneumoniae*, *iuc*3, septicaemia, whole-genome sequencing

## Abstract

*Klebsiella pneumoniae* (Kp), a ubiquitous pathogen found in diverse ecological niches, poses a threat to human and animal health. Hypervirulent Kp (hvKp) is concerning for its acquisition of virulence and antimicrobial resistance genes through plasmids. This study investigates hvKp as a cause of septicaemia in piglets in the Netherlands and examines the role of plasmids in virulence and host association. We collected 41 Kp isolates cultured from necropsies submitted from 15 different farms (2013–2020) and sequenced them using long-read sequencing. We identified sequence type (ST) 25 as the dominant Kp (67%, 10/15 farms) associated with septicaemia in pigs in the Netherlands. ST25 isolates displayed a hypervirulent profile, including the K2 hyper-capsule type and carried an *iuc*3 virulence plasmid. Further analysis revealed two ST25 clonal groups: CG25 and CG3804, a novel porcine clone. Multidrug resistance was identified in CG25 isolates from five pig farms. There was one colistin-resistant isolate carrying *mcr*-1 on a plasmid. Comparative genomic analysis was performed by including a large dataset of related publicly available Kp genomes from ST25 humans (*n*=230) and pigs (*n*=12) of all STs for phylogenetic and plasmid analysis. Pangenomic analysis revealed significantly higher *iuc*3 prevalence in global CG25 pig isolates (98%, 40/41) compared to humans (10%, 24/234) correlating with their enhanced virulence (scores 3–4 vs 0–1). The study highlights ST25 hvKp causing septicaemia in piglets in the Netherlands for the first time. Aerobactin lineage *iuc*3 on a plasmid is associated with infections in pigs and is responsible for an increased virulence score.

**Impact Statement**

This study addresses a critical knowledge gap in the molecular epidemiology of hypervirulent *Klebsiella pneumoniae* in animals, using comparative genomics to highlight the emergence of hypervirulent sequence type 25 Kp in pigs from the Netherlands. We compared these strains with global human and pig isolates, revealing the presence of *iuc*3 plasmids in pig isolates, which have also been identified in some human clinical isolates. This finding underscores a potential public health threat, as these plasmids may introduce hypervirulence through horizontal gene transfer. The detection of multidrug-resistant strains further amplifies this threat, with resistance genes potentially complicating treatment in both humans and animals.

## Data Summary

All the genomes have been submitted and achieved under the European Nucleotide Archive project ENA PRJEB72410.

## Introduction

*Klebsiella pneumoniae* (Kp) is a Gram-negative, rod-shaped bacterium that widely inhabits various ecological niches, including the human oral cavity and gastrointestinal tract, the digestive system of animals, soil and water [1]. It ranks among the top three causes of antimicrobial resistance-related deaths in humans worldwide [2] and remains a common nosocomial pathogen. The most well-known strain, classical *Klebsiella pneumoniae* (cKp), is responsible for the majority of hospital-acquired infections and is often multidrug resistant, posing a significant risk to immunocompromised individuals [3].

In recent decades, a new pathotype known as hypervirulent *Klebsiella pneumoniae* (hvKp) has emerged, primarily associated with community-acquired infections in humans but now becoming the dominant nosocomial pathogen in some health systems [4]. Unlike cKp, hvKp causes metastatic infections and is associated with high mortality rates. The first documented case of hvKp dates back to the mid-1980s when it was identified in healthy individuals in Taiwan, linked to pyogenic liver abscesses and septic endophthalmitis [5].

While hvKp strains were previously predominant in Asia and rarely resistant to antibiotics such as carbapenems, a recent report from the European Centre for Disease Prevention and Control highlighted the emergence of hvKp strains carrying carbapenemase genes in the European Union (EU) or the European Economic Area (EEA) countries [6]. Their risk assessment regarding the further spread of these hypervirulent strains concluded that the risk was high in healthcare settings. The rise of hvKp, particularly carbapenem-resistant hvKp, is concerning due to its association with higher morbidity and mortality [7]. Thus, it is crucial to accurately detect and characterize hvKp strains.

HvKp strains can be identified by combining both a phenotypic string test to examine the mucoid phenotype of bacterial colonies (referred to as the hypermucoviscous phenotype) and a molecular approach to detect mobile genetic element-encoded accessory genes, namely, yersiniabactin (*ybt*), aerobactin (*iuc*), colibactin (*clb*) and/or salmochelin (*iro*) [8]. Additionally, they are characterized by specific capsular polysaccharide antigens (K1, K2, K5, K20, K54 and K57), with K1 and K2 accounting for 70% of global hvKp cases [9]. Nassif *et al*. demonstrated that the existence of a ~180 kbp plasmid carrying aerobactin and its receptor protein in Kp strains was correlated with virulence and suggested that the siderophore aerobactin that scavenges host iron is an essential factor of pathogenicity in Kp. This suggests that the acquisition of virulence genes, typically through plasmids, has led to the emergence of hvKp [10].

Our understanding of the sources and circulation of hvKp strains and their plasmids in various ecological niches remains limited. A ‘One Health’ study conducted in the Italian city of Pavia scrutinized over 3,000 genomes from hospitals, pets, farms, livestock, plants and water sources. The findings indicate that humans serve as the primary reservoir of infections in hospitals, implying that the transmission of animal and environmental strains to humans resulting in nosocomial infections is limited [11]. Nevertheless, continuous monitoring of hvKp data from all available sources can provide insights into the prevalence, transmission, ecology and plasmid evolution of hvKp. Currently, data on hvKp strains other than human clinical isolates are limited, emphasizing the need to identify and report global problem clones in animals and the environment.

In swine, septicaemia cases attributed to Kp are rare. Outbreaks in piglets have only been reported in Australia [12] and the UK [13], and most cases are sporadic in the UK [11]. These studies reported that similar septicaemia outbreaks in piglets were associated with Kp sequence type (ST) 25.

In this study, we report our passive surveillance data of septicaemia cases caused by Kp in swine in the Netherlands for the first time. In addition, we describe the molecular epidemiology of these cases and determine their genetic relatedness to human and other pig strains and investigate the role of plasmids in virulence and resistance.

## Methods

This retrospective study in the Netherlands investigated Kp cultured from necropsies of piglets submitted to Royal Gezondheidsdienst voor Dieren (GD) Animal Health Service, by 15 different pig farms experiencing sudden deaths in piglets (2013–2020). Spleen, brain and lung tissues were collected from affected piglets during post-mortem examination (one to six isolates per submission per farm). To minimize overrepresentation and sampling bias, proportions were calculated based on the farm level (*n*=15). Bacterial species were identified using MALDI-TOF MS (MALDI Biotyper, Bruker Daltonics GmbH and Co. KG, Bremen, Germany). A string test was used to determine a hypermucoviscous phenotype (>5 mm stretch of the colony when the colony was picked up with a loop). DNA isolation was performed using the DNeasy® UltraClean® Microbial kit (Qiagen GmbH, Hilden, Germany) according to the manufacturer’s protocol. The isolated DNA was quantified with the Qubit dsDNA High Sensitivity assay using a Qubit 4 Fluorometer. Long-read sequencing libraries were prepared with the Rapid Barcoding Kit 96 SQK-RBK110.96, and the barcoded samples were sequenced on a MinION device using flow cell type R9.4.1 (FLO-MIN 106D) (Oxford Nanopore, Oxford, UK). All sequences were trimmed with TrimGalore v0.4.4 [14], assembled using Flye v2.9 with default parameters [15] and annotated with Prokka v1.11 [16]. The quality of genomes was checked with CheckM v1.1.3 [17], and only genomes with a contamination threshold of <5% and a completeness threshold of >94% were included in the analysis (Table S2, available in the online Supplementary Material). The comparative genome analysis was performed using Roary v3.13.0 with a minimum of 95% identity [18]. The genomes were analysed using Kleborate v2.2.0 [19] to detect virulence and resistance genes, whereas Kaptive Web v1.3.0 using its databases v3.0.0b6 was used to type K and O-loci [20,21]. The virulence score (0–5) was determined by the Kleborate tool as follows: 0 = none present; 1 = yersiniabactin *ybt* only; 2 = colibactin *clb* without *aerobactin iucC* (regardless of *ybt*); 3 = aerobactin *iucC* only; 4 = aerobactin *iucC* and yersiniabactin *ybt* without colibactin *clb*; and 5 *= iucC*, *ybt* and *clb*.

### Core-genome multi-locus sequence typing and LIN code sub-lineage analysis

Core-genome multi-locus sequence typing (cgMLST) was performed using Kleborate v2.2.0 with the Kp Institut Pasteur cgMLST scheme via the BIGSdb platform [22,23]. Thresholds for allele calling and cluster definition followed the scheme’s default parameters (≥95% locus completeness). To resolve fine-scale population structure within ST25, hierarchical clustering of SNPs was used to assign the LIN code, clonal group (CG), and sublineage (SL), as implemented in Kleborate [19,23].

### Plasmid typing and analysis

For plasmid analysis, Bandage v0.8.1 was used to check the assembly graphs of plasmid contigs [24], resulting in 35 closed genomes. Plasmid contigs were then extracted using Geneious Prime 2022.0.2 and annotated with Prokka v1.11 [16]. These plasmid contigs were analysed using Roary v3.13.0 [18], PlasmidFinder v2.1 [25] and ResFinder v4.0 [26].

The representative plasmid contig containing aerobactin (21S00310-1contig_3) was visualized using the KpVR web-based tool v1.0 (*https://db-mml.sjtu.edu.cn/KpVR/*) to analyse virulence plasmids [27]. This tool provides replicon typing, virulence/resistance gene annotation and visualization of plasmid architecture. KpVR uses a curated database (repDB) that includes *IncFIBK* subtypes and integrates *AbST/SmST* schemes for *iuc* and *iro* lineage typing. For this study, all *IncFIBK* plasmids were analysed using KpVR, which performs nt identity/coverage-based searches to assign replicons (e.g. *IncFIB*[*K*]/*IncFII*), maps virulence modules (e.g. *iucABCD-iutA*) and generates graphical plasmid overviews. The tool’s output includes replicon alleles, type four secretion system (T4SS) clusters and co-localized resistance genes (e.g. heavy metal resistance loci). The plasmid pCY814036-iucA (GenBank accession no. CP093152) [28], identified from an ST25 hvKp strain isolated from a Chinese patient, was included in the plasmid analysis. This plasmid carries both multidrug-resistant (MDR) genes and the virulence-associated *iuc* operon on a single conjugative plasmid. We included this particular plasmid from China as it serves as a reference for ST25 aerobactin lineage *iuc*3, allowing us to compare and determine if our *iuc*3 plasmids from pig isolates share similarities with the *iuc* plasmid isolated from humans.

### Comparative genome analysis

In addition, we analysed a large collection of Kp ST25 genomes both sequenced in this study (*n*=41) and reported previously. The collection included ST25 genomes of human origin (*n*=234) and pig origin (*n*=12) from public databases (accessed on 15 November 2022) curated in Pathogenwatch [29] and Bacterial and Viral Bioinformatics Resource Center (BV-BRC) [30] to examine global emergence and dissemination of ST25 hvKp isolates. Only genomes sequenced in this study (*n*=41) have been submitted and achieved under project ENA PRJEB72410.

### Phylogenetic tree construction

**Fig. 1.**
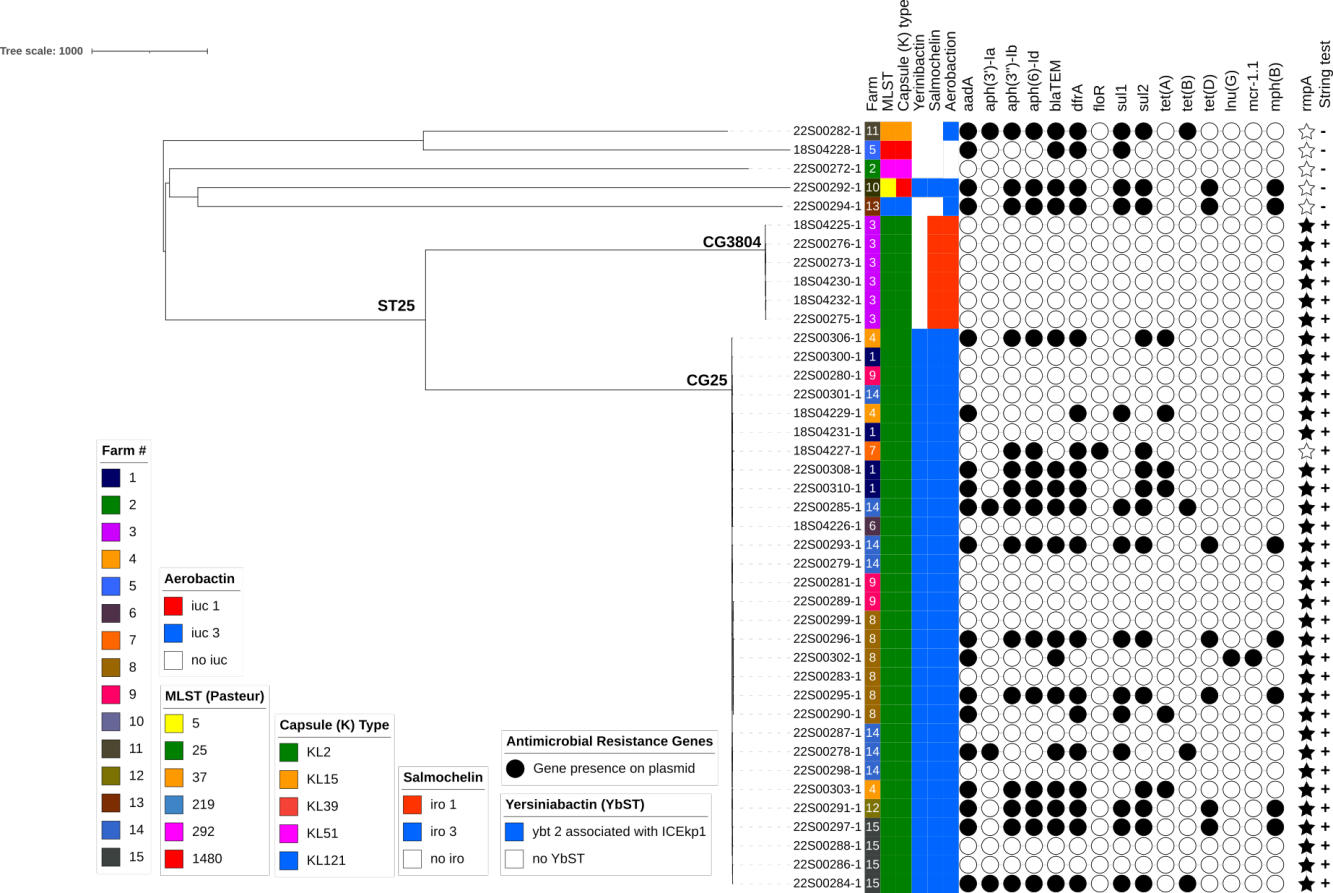
Phylogenetic tree and metadata of sepsis-causing Kp in pigs from the Netherlands (2013–2020). Black circles represent genes present on plasmids. Black stars represent the presence of rmpA genes. For the string test column, + means positive for string test, and – means negative for string test. *blaSHV*, beta-lactam resistance; *fosA*, fosfomycin resistance; *oqxA*, *oqxB*, efflux pump; *aadA*, *aph(3′)-la*, *aph(3′)-lb, aph(3′)-ld*, *aph(6′)ld*, aminoglycoside resistance; *blaTEM*, beta-lactam resistance; *dfrA*, trimethoprim resistance; *floR*, chloramphenicol resistance; *sul1*, *sul2*, sulphonamide resistance; *tet(A)*, *tet(B)*, *tet(D*), tetracycline resistance; *lnu(G*), lincosamide resistance; *mcr-1.1*, colistin resistance; *mph*(*B*), macrolide resistance; *iuc3, iuc1*, aerobactin synthesis; *iro1*, *iro3,* Salmochelin; *ybt,* Yersiniabactin. Tree scale: 1,000 means that branch lengths are scaled by a factor of 1,000.

**Fig. 2.**
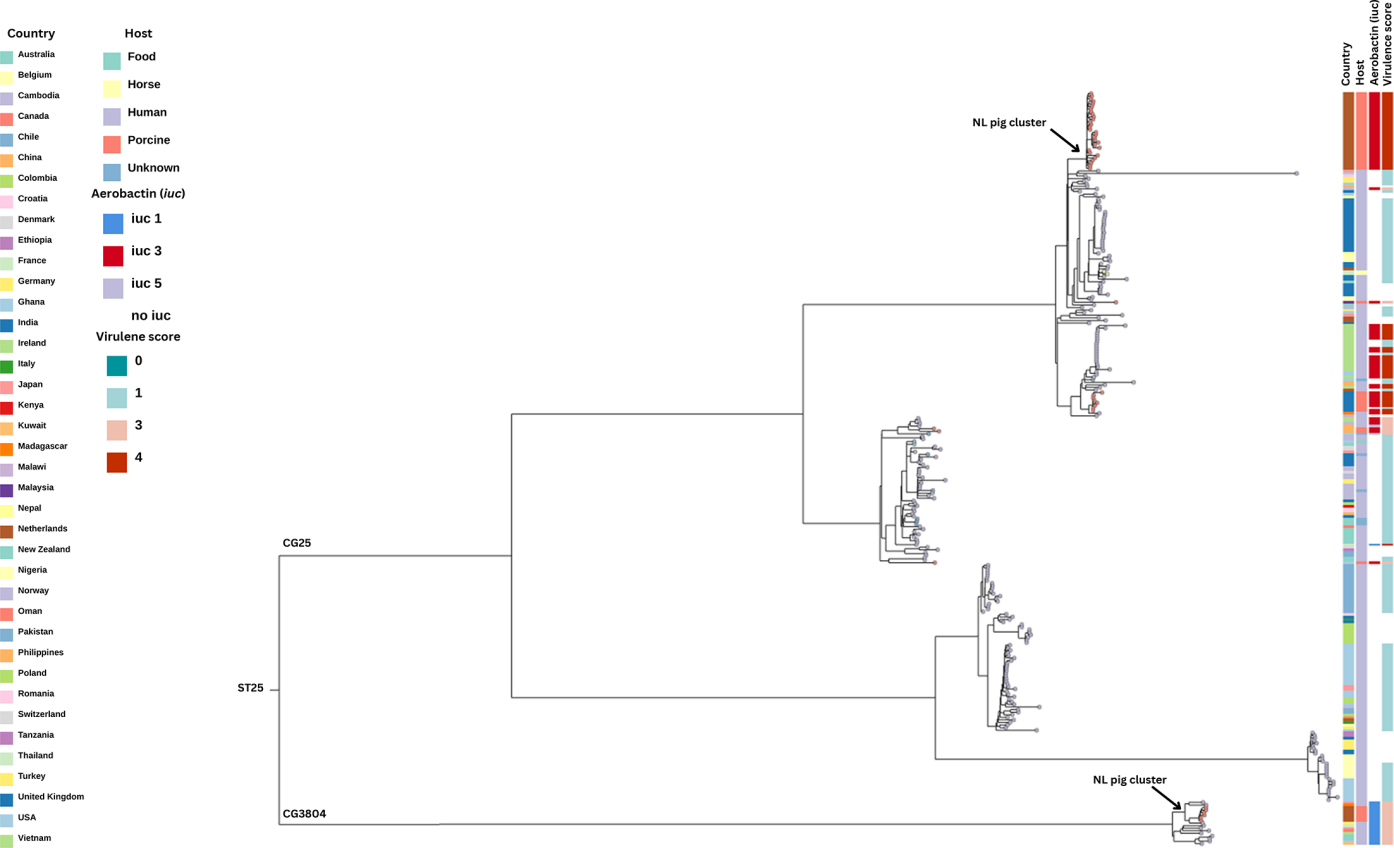
Global phylogeny of ST25 isolates from different hosts and different countries (leaf node tip colour represents the host [isolation source; NL (the Netherlands) pig cluster isolates represent the isolates from this study].

The phylogenetic tree (Fig. 1) was constructed using Pathogenwatch (https://pathogen.watch), and Interactive Tree of Life (iTOL) v6.0 [31] was utilized to visualize the metadata of the genomes in a mid-rooted phylogenetic tree. The global phylogenetic tree (Fig. 2) was constructed using a newick tree output from Pathogenwatch and annotated using Microreact [32]. A plasmid tree (Fig. S1) was constructed using RAxML v8.2.4 [33] with the BINCAT model for binary gene presence–absence data, 100 bootstrap replicates and rapid bootstrapping followed by maximum likelihood tree search. The minimum spanning tree (MST) (Fig. S2) was generated using PhyloViz v2.0 [34] by applying the goeBURST algorithm [35] to a core genome SNP alignment of Kp isolates from this study produced with Parsnp v1.7 [36] and SNP-sites [37], with nodes representing strains and edge lengths proportional to the number of allelic differences in the core genome.

## Results

### Study population

**Fig. 3.**
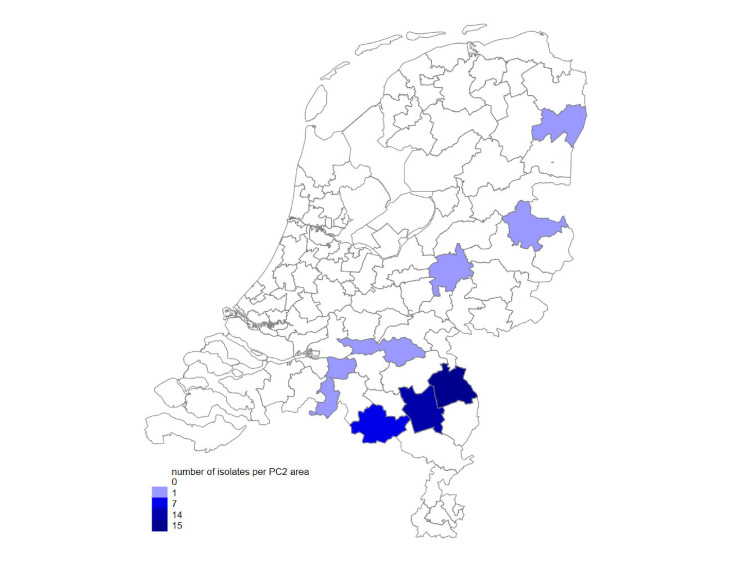
The distribution map of Kp isolates cultured from pigs submitted for post-mortem examination (*n*=41) from pig farms in the Netherlands (2013–2020).

A total of 41 Kp isolates were recovered from 15 different farms that submitted pigs for post-mortem examination due to sudden pig deaths (2013–2020). The median age of piglets was 2 weeks (interquartile range: 1 week, range: 1–104 weeks). Septicaemia cases were confirmed using a case definition described in a previous study [38]: sudden death in pigs with lesions consistent with septicaemia and pure/predominant growth of Kp isolated from internal organs of diseased animals. Most cases were submitted from indoor farms in the southern part of the Netherlands where pig density is high. Fig. 3 depicts the spatial distribution of Kp*-*associated septicaemia cases by postal code region. Table 1 summarizes isolate information and phenotypic and genotypic characteristics.

**Table 1.** Summary of Kp isolates associated with septicaemia in the Netherlands, 2013–2020

Isolate	Age (weeks)	Source from pigs	Farm	Year	Month	MLST	cgMLST	String test	Virulence
22S00272-1	na	Lung	2	2013	January	ST292	CG292	Negative	3
18S04225-1	na	Brain	3	2015	July	ST25	CG3804	Positive	3
18S04230-1	2	Brain	3	2015	August	ST25	CG3804	Positive	3
18S04232-1	na	Brain	3	2015	March	ST25	CG3804	Positive	3
22S00273-1	na	Spleen	3	2015	August	ST25	CG3804	Positive	3
22S00275-1	2	Brain	3	2015	August	ST25	CG3804	Positive	3
22S00276-1	na	Spleen	3	2015	August	ST25	CG3804	Positive	3
18S04231-1	2	Brain	1	2017	August	ST25	CG25	Positive	4
22S00308-1	2	Brain	1	2017	August	ST25	CG25	Positive	4
22S00310-1	na	Submitted culture	1	2017	September	ST25	CG25	Positive	4
18S04229-1	3	Spleen	4	2017	July	ST25	CG25	Positive	4
22S00303-1	3	Spleen	4	2017	July	ST25	CG25	Positive	4
22S00306-1	3	Spleen	4	2017	July	ST25	CG25	Positive	4
18S04228-1	na	Spleen	5	2017	August	ST1480	na	Negative	3
18S04226-1	3	Brain	6	2017	August	ST25	CG25	Positive	4
18S04227-1	3.14	Spleen	7	2017	October	ST25	CG25	Positive	4
22S00283-1	2	Joint	8	2018	June	ST25	CG25	Positive	4
22S00290-1	2	Spleen	8	2018	June	ST25	CG25	Positive	4
22S00295-1	2	Spleen	8	2018	June	ST25	CG25	Positive	4
22S00296-1	2	Spleen	8	2018	June	ST25	CG25	Positive	4
22S00299-1	2	Spleen	8	2018	June	ST25	CG25	Positive	4
22S00302-1	2	Joint	8	2018	June	ST25	CG25	Positive	4
22S00280-1	2	Brain	9	2018	August	ST25	CG25	Positive	4
22S00281-1	2	Brain	9	2018	August	ST25	CG25	Positive	4
22S00289-1	2	Spleen	9	2018	June	ST25	CG25	Positive	4
22S00279-1	na	Spleen	14	2018	November	ST25	CG25	Positive	4
22S00285-1	na	Liver	14	2018	November	ST25	CG25	Positive	4
22S00298-1	na	Brain	14	2018	November	ST25	CG25	Positive	4
22S00292-1	2	Brain	10	2019	March	ST5	CG5	Negative	4
22S00282-1	6	Spleen	11	2019	June	ST37	CG37	Negative	3
22S00291-1	na	na	12	2019	December	ST25	CG25	Positive	4
22S00300-1	1	Brain	1	2020	August	ST25	CG25	Positive	4
22S00294-1	104	na	13	2020	July	ST219	CG107	Negative	3
22S00278-1	na	Brain	14	2020	April	ST25	CG25	Positive	4
22S00287-1	na	Brain	14	2020	April	ST25	CG25	Positive	4
22S00293-1	na	Brain	14	2020	April	ST25	CG25	Positive	4
22S00301-1	na	Brain	14	2020	April	ST25	CG25	Positive	4
22S00286-1	na	Ascites	15	2020	August	ST25	CG25	Positive	4
22S00297-1	na	Mesentery	15	2020	August	ST25	CG25	Positive	4
22S00284-1	na	Mesentery	15	2020	August	ST25	CG25	Positive	4
22S00288-1	na	Ascites	15	2020	August	ST25	CG25	Positive	4

na, not applicable. The virulence score (0–5) was determined by the Kleborate tool as follows: 0, none present; 1, ybt only; 2, clb without iucC (regardless of ybt); 3, iucC only; 4, iucC and ybt without clb; 5, iucC, ybt and clb.

### Genomic information of ST25 hypervirulent strains in the Netherlands

Molecular characterization of the Kp isolates revealed that multi-locus sequence type (MLST) ST25 was dominant in the majority of farms (67%, 10/15) in this study. The remaining five farms showed diverse MLST types: ST5 (farm 10), ST37 (farm 11), ST219 (farm 13), ST292 (farm 2) and ST1480 (farm 5). This variation in STs corresponded to distinct clustering patterns based on the origin of isolates, including the farm and geographic region, capsule type (K) and hypervirulent determinants such as aerobactin, salmochelin and yersiniabactin (Fig. 1). ST25 isolates were not genetically closed to other STs as indicated by the MST shown in Fig. S2.

Unlike isolates with different STs, ST25 isolates displayed hypervirulence characteristics, including the presence of the K2 polysaccharide capsule type and virulence-associated genes encoding aerobactin (*iuc*), yersiniabactin (*ybt2*) and salmochelin (*iro3*). Kaptive results predicated a capsule null phenotype due to truncations in critical genes such as *wzi* and *wzy*. The ‘capsule null’ prediction in ST25 isolates, despite K2 typing, indicates capsule-deficient *Klebsiella* lineages. All ST25 isolates demonstrated a hypermucoviscous phenotype, confirmed by positive results in the phenotypic string test (Table 1). All non-ST25 isolates displayed a negative string test, indicating the absence of hypermucoviscosity. Non-ST25 isolates did not carry any aerobactin, salmochelin and yersiniabactin genes except one ST219 genome. Kleborate analysis showed two distinct clonal groups of ST25: CG25 and CG3804. These clusters align with LIN code SLs SL25 and SL3804 (Table S1), respectively. All isolates within CG25 exhibited a virulence score of 4, and those within CG3804 had a virulence score of 3. As seen in Fig. 1, all CG25 isolates, CG5 and CG 219 carried *iuc* allele *iuc3*, whereas CG3804 isolates carried *iuc1*. In terms of antibiotic resistance, bla*SHV* (providing intrinsic resistance to ampicillin and other *β*-lactams), the *fosA* gene (conferring fosfomycin resistance) and the *OqxAB* gene coding for efflux pump, responsible for MDR, are encoded on the chromosome in all Kp isolates.

MDR, defined as not susceptible to three distinct antibiotic groups or more, was identified in CG25 isolates from five farms (farms 1, 2, 8, 9 and 15) carrying resistance genes of streptomycin, spectinomycin, tetracycline and trimethoprim-sulphonamide (Fig. 1). A notable finding was the detection of one colistin-resistant hvKp CG25 isolate harbouring the *mcr*-1 gene on a plasmid (22S000291-1, farm ID 12). No carbapenemase genes were detected in any isolate. Antimicrobial resistance genes (ARGs) conferring resistance to aminoglycoside, trimethoprim, chloramphenicol, sulfamethoxazole, colistin, macrolide and tetracycline were also encoded on plasmids.

### Hypervirulence gene presence on plasmids

Complete genome analysis was conducted to precisely determine the location of virulence genes, whether on plasmids or the chromosome. Plasmid typing revealed a clustering pattern based on *Inc* types, showcasing a variety of plasmid types associated with virulence genes and ARGs (Fig. S1).

**Fig. 4.**
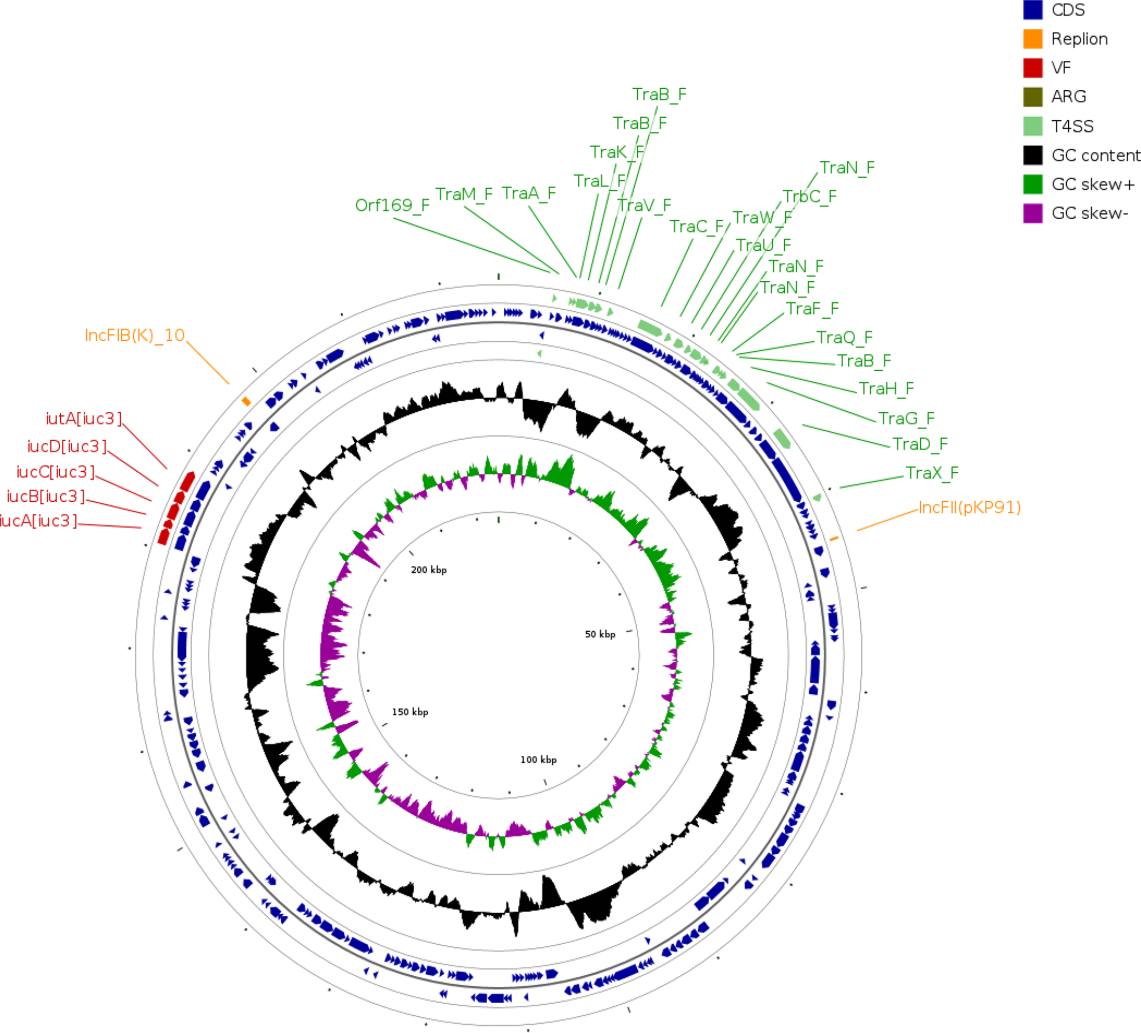
Aerobactin virulence *iuc*3 plasmid carrying *iuc*3 genes from CG25 hvKp isolates from piglets in the Netherlands (21S00310-1contig_3).

Plasmid analysis confirmed that CG25 hvKp isolates had acquired aerobactin, encoded by the *iuc3* lineage of the aerobactin locus operon, named *iuc*3 plasmid in this study. The aerobactin genes responsible for Kp’s iron scavenging from the host, *iucABCD* and *iutA* genes, were located on this plasmid in all CG25 isolates. This plasmid type belonged to the F plasmid incompatibility groups IncFIB(K) and IncFII (Fig. 4). Furthermore, it contains the Fec-like iron(III) dicitrate transport system, a T4SS, as well as several heavy metal resistance genes including arsenic and copper.

### Phylogeny of global ST25 isolates from humans and pigs

As virulence and antimicrobial resistance in pig isolates appear to be associated with specific plasmids such as *iuc*3 plasmid in ST25 isolates, we added publicly available ST25 Kp isolates mostly from human and pig sources to our analysis. The global phylogenetic tree of ST25 reveals clear differences between Dutch hvKp isolates and those from other parts of the world (Fig. 2). This pangenome analysis highlights two clonal groups: CG25 and CG3804. Within the CG25 lineage, two main clades are present. One is comprised of human isolates not carrying aerobactin, while the other includes pig and human isolates that carry aerobactin. In general, pig isolate clusters are associated with their geographical origin. Dutch pig isolates of the CG25 lineage were genetically different from the other pig isolates originated from the UK, China, Malaysia and the USA (Fig. 2). In comparison to our Dutch study isolates, pig isolates from China and the UK are hvKp carrying *iuc3* plasmids. It is noteworthy that a similar iuc3 plasmid has been identified in MDR ST25 hvKp isolates [CY814036, and the plasmid pCY814036-iucA (GenBank accession no. CP093152) [28]] from neonatal children in China (Fig. S1). Despite sharing the same ST, the majority of CG25 isolates (84.1%, 197/234) from humans display lower virulence scores (0–1), whereas all CG25 isolated from pigs and CG3804 isolates from both humans and pigs had higher virulence score [3,4]. On the other hand, *iuc*3 prevalence was higher in global pig isolates (98%, 40/41) compared to humans (10%, 24/234) correlating with their enhanced virulence (scores 3–4 vs 0–1). The detailed description of global ST25 isolates is summarized in Table S1.

## Discussion

This is the first retrospective study describing hvKp as the causative agent of septicaemia in piglets in the Netherlands. Similar to previous septicaemia outbreaks in the UK [13] and Australia [12], our genomic analysis revealed that Dutch pig isolates shared the same ST25. We observed the presence of two distinct clusters within ST25, namely, CG25 and CG3804. Dutch CG25 pig isolates formed a cohesive and genetically distinct cluster from other pig and human isolates, indicating the emergence of an animal species-specific CG25 hvKp in the Netherlands. CG3804 appeared to be a novel clone in pigs, clustering alongside 11 human isolates of CG3804 from different regions including Asia, Europe, America, Africa and Australia, suggesting a clonal spread of CG3804 worldwide. All CG3804 isolates carried aerobactin lineage *iuc*1 and *iro1* (salmochelin) virulence genes on plasmids, while CG25 isolates carried aerobactin lineage *iuc3* on plasmids with IncFIB(K) and IncFII replicon. Similarly, Lam *et al.* [8] demonstrated that *iuc3* plasmids were present in CG25, CG35 and CG231 isolates. Consistent with our findings, the *iuc3* lineage was linked to IncFIB(K) and IncFII replicons [8]. In addition, CG25 and CG3804 isolates had virulence scores of 4 (aerobactin and yersiniabactin) and 3 (aerobactin only), respectively. This difference in virulence score between CG25 and CG3804 isolates can be attributed to the presence of a distinct *iuc*3 plasmid in all pig CG25 isolates and *iuc*1 plasmids in CG3804 isolates. Despite sharing the same CG25, in comparison to human isolates, all pig isolates contained *iuc3* virulence plasmids. The pangenomic analysis showed an association between *iuc3* virulence plasmids and host, as CG25 human isolates lacking *iuc3* plasmids exhibited lower virulence score (0–1), while mainly CG25 pig isolates containing the presence of *iuc*3 increased the virulence score to 4.

A recent study conducted in Norway reported a high frequency of *iuc3* plasmids in Kp found within healthy fattening pig populations in the country [39]. Additionally, another study from Italy highlighted the presence of *iuc*3 plasmids in raw pork meat and its processing environment within two artisanal ready-to-eat food productions [40]. Subsequently, their findings revealed strong similarities of *iuc*3-carrying plasmids found in the artisanal food production chain and those present in Kp strains isolated from both humans and pig livestock originating from the same geographical area. This finding suggests the possibility of a transfer of virulence plasmids across strains found in different host species such as humans and pigs.

It is well established that aerobactin plays a crucial role in the pathogenicity of extracellular pathogens, as observed in septicaemia and urinary tract infections caused by *Escherichia coli* and Kp [41]. Previous studies have highlighted that biomarkers including salmochelin, *iro*, and aerobactin, *iuc*ABCD, as well as a siderophore enterobactin receptor, *fep*A, and a ferric aerobactin receptor, *iut*A, are associated with liver invasion in human isolates. Interestingly, *iuc*3-harbouring plasmids are notably less present in human Kp isolates. Less than 1% of healthy human isolates in Norway [39] and 10% of global CG25 human isolates in the current study carried *iuc*3 plasmids. A European study on carbapenem-resistant Kp (CRKP; EuSCAPE) also showed that only 0.7% of human isolates (11 out of 1,505 CRKP human isolates) carried *iuc*3 plasmids [42]. Our results align with a study conducted in Germany, which found that aerobactin of lineage *iuc3* was exclusively detected in Kp isolates from domestic pigs and wild boars within the country [43]. These findings suggest that *iuc3* plasmids are commonly found in pigs and hvKp CG25 may have invasive potential, causing septicaemia in pigs and invasive infections in humans, particularly when the aerobactin *iuc3* plasmid is present.

We could not determine when and how hvKp strains from pigs acquired *iuc*3 plasmid due to our limited dataset. It is still unclear whether hvKp strains are transmitted between different hosts or if plasmids are transferred between strains instead. This information can be helpful in elucidating the mechanisms behind the evolution of virulence plasmids.

Convergent plasmids, a plasmid that is formed by fusion of two or more smaller plasmids, with MDR and hvKp determinants have been found in China where the same CG25 and the same plasmid *iuc*3 converged with multiple ARGs were recovered from neonatal sepsis case in children [44]. These fusion plasmids are indicative of MDR hypervirulent isolates (e.g. a single plasmid carrying *iuc* virulence genes together with ESBL and/or carbapenemase genes). In our study, we did not identify convergent plasmids in the Netherlands. Nevertheless, some CG25 isolates in our study displayed MDR, carrying resistance genes conferring resistance to aminoglycosides, tetracycline and trimethoprim-sulphonamide on a different plasmid. It is important to note that one *mcr*-1 plasmid-mediated colistin-resistant hvKp isolate was recovered from pigs in the Netherlands. Even though convergent plasmids have not yet been found in the Netherlands, MDR hvKp circulating in food-producing animals should raise concerns and call for early reporting and monitoring. Given the increasing prevalence of high-risk clones such as ST11 or ST23 in hospital settings in EU/EEA regions, it is also important to monitor high-risk clones occurring in animals and the environment [7]. In this context, reporting unusual cases from animals plays a pivotal role in early detection. In the Netherlands, early detection programmes are in place for unusual cases, and when combined with genomic analyses like in this study, they provide a framework for genomic surveillance of emerging hvKp and virulence and resistance-associated plasmids.

On the other hand, our study has several limitations. Firstly, there is geographic sampling bias due to submissions predominantly from locations with high pig density in the Netherlands. Moreover, there are variations in the number of pigs submitted per farm and variation in the number of isolates per submission, with some submitting only one animal while others submitted up to six animals, potentially skewing the dataset. To reduce over-representativeness and sampling bias, we presented our data on a farm level instead of isolate level. Since this is a retrospective study based on diagnostic samples, we did not have clinical data and epidemiological data including on-farm risk factor data, which is another constraint on the interpretation of our findings. Moreover, there were no isolates collected from healthy animals to compare, and further research should look into whether healthy pigs carry the same hvKp strain. Despite the inherent limitations of a retrospective passive surveillance study, our research provides valuable information on the molecular epidemiology of hvKp, enhancing our understanding of its plasmid ecology and genetic diversity.

To conclude, our findings suggest that the presence of plasmids carrying *iuc*3 lineage is associated with pig as a host. Further research is needed to understand the role of plasmids encoding *iuc*3 in hvKp virulence and transmission. This information will be crucial for understanding the ecology and epidemiological dynamics of hvKp from different sources.

## Supplementary material

10.1099/mgen.0.001388 Uncited Supplementary Material 1.

10.1099/mgen.0.001388 Uncited Supplementary Material 2.

10.1099/mgen.0.001388 Uncited Supplementary Material 3.

## Abbreviations

ARGs: antimicrobial resistance genes
cgMLST: core-genome multi-locus sequence typing
cKp: classical *Klebsiella pneumoniae*
CRKP: carbapenem-resistant Kp
EEA: European Economic Area
EU: European Union
hvKp: hypervirulent *Klebsiella pneumoniae*
Kp: Klebsiella pneumoniae
MDR: multidrug resistance
MLST: multi-locus sequence type
MST: minimum spanning tree
SL: sublineage
ST: sequence type
T4SS: type four secretion system
