# Supplementary material for: Molecular epidemiology and emergence of sequence type 25 hypervirulent Klebsiella pneumoniae in pigs in the Netherlands (2013–2020): a global comparative analysis with human and pig isolates
Source: Microb Genom. 2025 Apr 23;11(4):001388. doi: 10.1099/mgen.0.001388 (PMC12018708; doi:10.1099/mgen.0.001388)
Supplement: Uncited Supplementary Material 1. [file mgen-11-01388-s001.pdf]

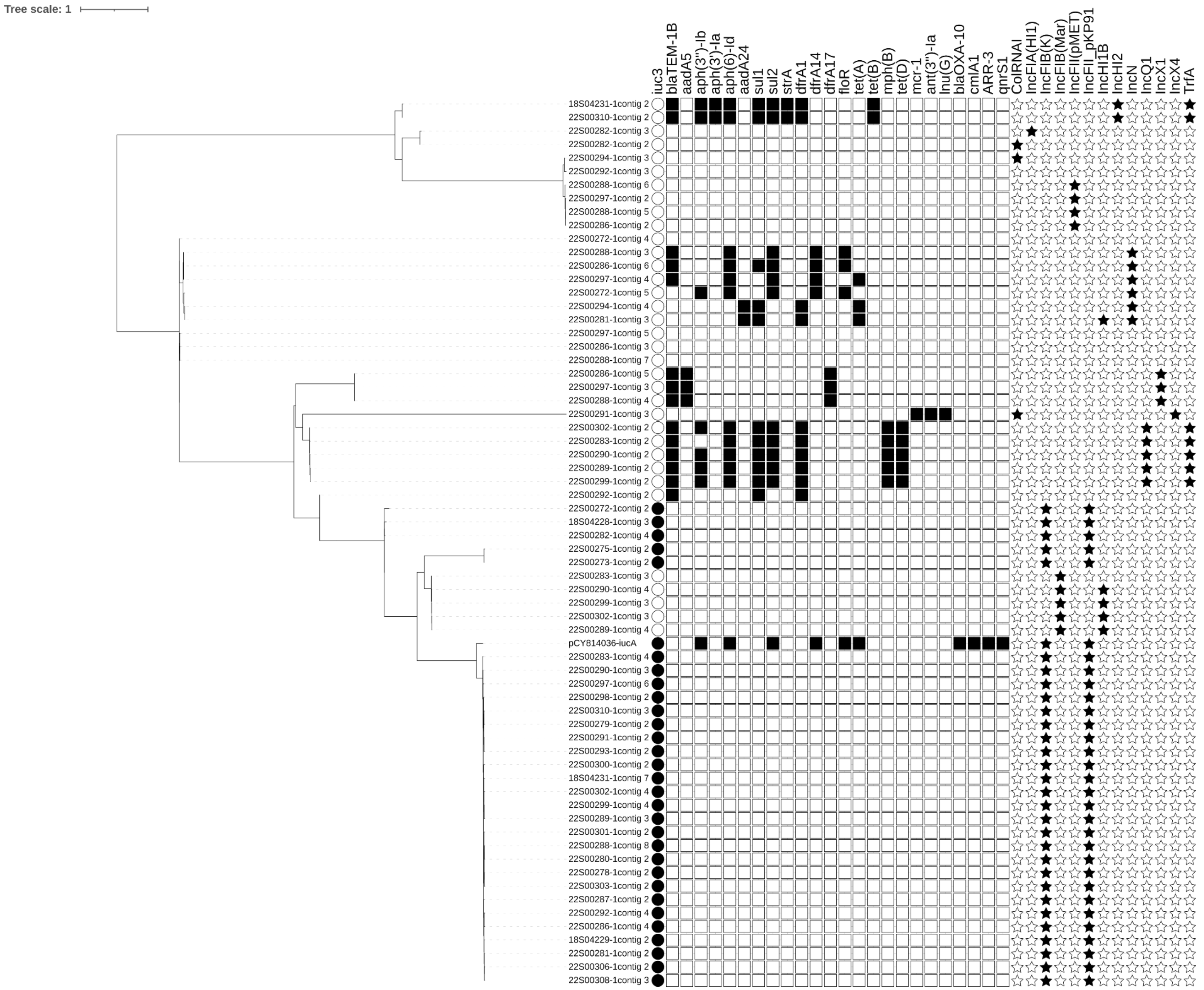

Tree scale: 1
iuc3
blaTEM-1B
aadA5
aph(3'')-Ib
aph(3')-Ia
aph(6)-Id
aadA24
sul1
sul2
strA
dfrA1
dfrA14
dfrA17
floR
tet(A)
tet(B)
mph(B)
tet(D)
mcr-1
ant(3'')-Ia
lnu(G)
blaOXA-10
cmlA1
ARR-3
qnrS1
ColRNAI
IncFIA(HI1)
IncFIB(K)
IncFIB(Mar)
IncFII(pMET)
IncFII_pKP91
IncHI1B
IncHI2
IncN
IncQ1
IncX1
IncX4
TrfA
18S04231-1contig 2
22S00310-1contig 2
22S00282-1contig 3
22S00282-1contig 2
22S00294-1contig 3
22S00292-1contig 3
22S00288-1contig 6
22S00297-1contig 2
22S00288-1contig 5
22S00286-1contig 2
22S00272-1contig 4
22S00288-1contig 3
22S00286-1contig 6
22S00297-1contig 4
22S00272-1contig 5
22S00294-1contig 4
22S00281-1contig 3
22S00297-1contig 5
22S00286-1contig 3
22S00288-1contig 7
22S00286-1contig 5
22S00297-1contig 3
22S00288-1contig 4
22S00291-1contig 3
22S00302-1contig 2
22S00283-1contig 2
22S00290-1contig 2
22S00289-1contig 2
22S00299-1contig 2
22S00292-1contig 2
22S00272-1contig 2
18S04228-1contig 3
22S00282-1contig 4
22S00275-1contig 2
22S00273-1contig 2
22S00283-1contig 3
22S00290-1contig 4
22S00299-1contig 3
22S00302-1contig 3
22S00289-1contig 4
pCY814036-iucA
22S00283-1contig 4
22S00290-1contig 3
22S00297-1contig 6
22S00298-1contig 2
22S00310-1contig 3
22S00279-1contig 2
22S00291-1contig 2
22S00293-1contig 2
22S00300-1contig 2
18S04231-1contig 7
22S00302-1contig 4
22S00299-1contig 4
22S00289-1contig 3
22S00301-1contig 2
22S00288-1contig 8
22S00280-1contig 2
22S00278-1contig 2
22S00303-1contig 2
22S00287-1contig 2
22S00292-1contig 4
22S00286-1contig 4
18S04229-1contig 2
22S00281-1contig 2
22S00306-1contig 2
22S00308-1contig 3

Supplementary Figure 1: Plasmid tree with the presence and absence of virulence genes, resistance genes, and plasmid replicons.

The tree illustrates the phylogenetic relationships among plasmids, with **pCY8Y1036-iucA** plasmid highlighted as a convergent ST25 plasmid from China. The presence or absence of genes is indicated as follows: filled shapes indicate the presence of a gene, unfilled shapes indicate the absence of a gene, circles represent virulence genes, squares represent resistance genes, stars represent plasmid replicons.

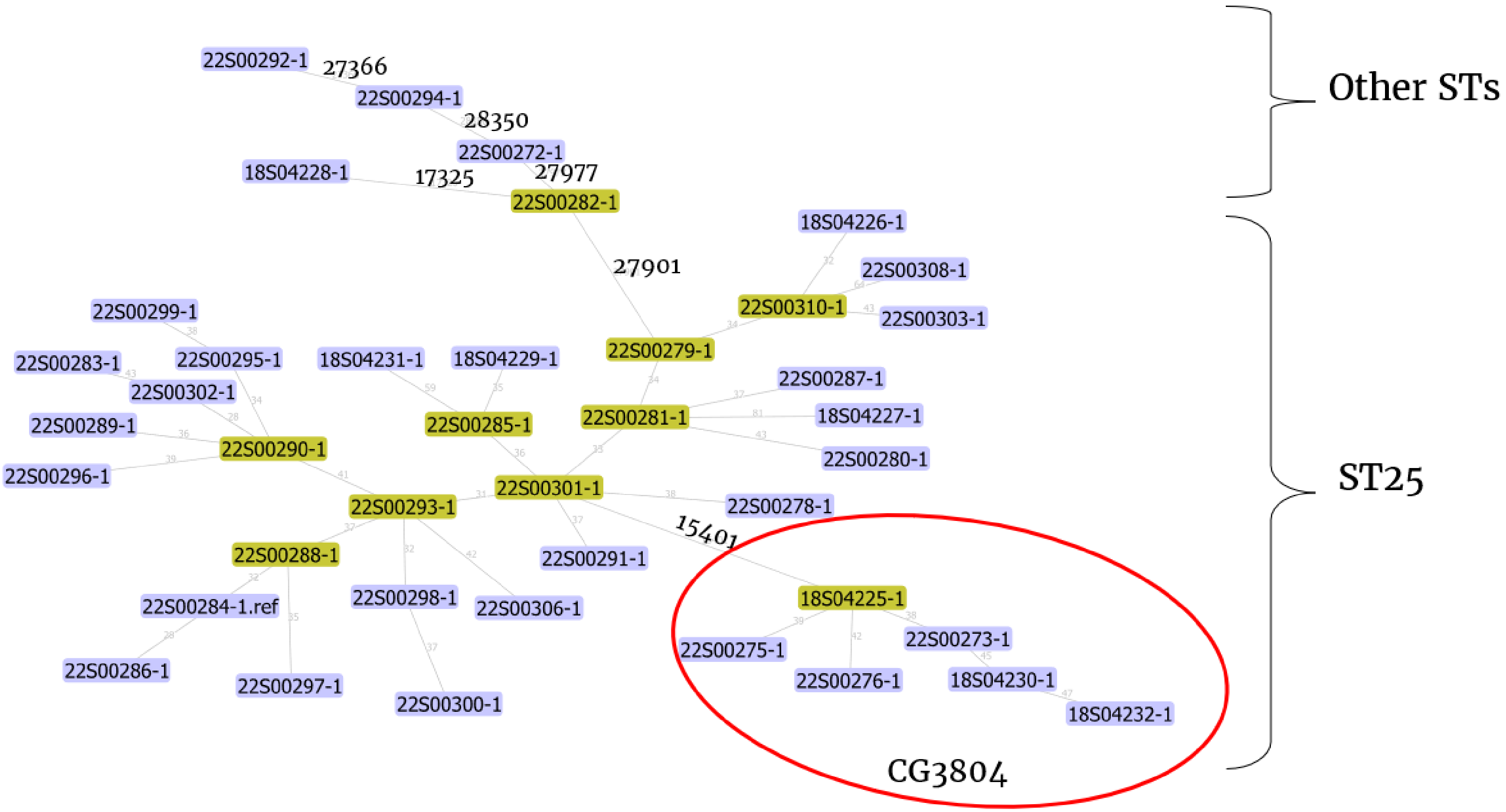

Supplementary Figure 2: Minimum spanning tree illustrating core genome SNP differences between sequence type 25 (ST25) and other sequence types (STs) of *Klebsiella pneumoniae* (Kp) isolates from pigs in the Netherlands, generated with PhyloViz using the goeBURST algorithm. The red circle highlights CG3804 isolates; numbers adjacent to branches indicate SNP distances between isolates.
